# Circulating levels of GDF15 in patients with myalgic encephalomyelitis/chronic fatigue syndrome

**DOI:** 10.1186/s12967-019-02153-6

**Published:** 2019-12-04

**Authors:** A. Melvin, E. Lacerda, H. M. Dockrell, S. O’Rahilly, L. Nacul

**Affiliations:** 1 MRC Metabolic Diseases Unit, Wellcome Trust-MRC Institute of Metabolic Science, University of Cambridge, Addenbrookes Treatment Centre, Cambridge, CB2 0QQ UK; 2 Department of Clinical Research, Faculty of Infectious and Tropical Diseases, London School of Hygiene & Tropical Medicine, London, WC1E 7HT UK; 3 Department of Immunology and Infection, Faculty of Infectious and Tropical Diseases, London School of Hygiene & Tropical Medicine, London, WC1E 7HT UK

**Keywords:** GDF15, Myalgic encephalomyelitis, Chronic fatigue syndrome

## Abstract

**Background:**

Myalgic encephalomyelitis/chronic fatigue syndrome (ME/CFS) is a debilitating condition characterised by fatigue and post-exertional malaise. Its pathogenesis is poorly understood. GDF15 is a circulating protein secreted by cells in response to a variety of stressors. The receptor for GDF15 is expressed in the brain, where its activation results in a range of responses. Among the conditions in which circulating GDF15 levels are highly elevated are mitochondrial disorders, where early skeletal muscle fatigue is a key symptom. We hypothesised that GDF15 may represent a marker of cellular stress in ME/CFS.

**Methods:**

GDF15 was measured in serum from patients with ME/CFS (n = 150; 100 with mild/moderate and 50 with severe symptoms), “healthy volunteers” (n = 150) and a cohort of patients with multiple sclerosis (n = 50).

**Results:**

Circulating GDF15 remained stable in a subset of ME/CFS patients when sampled on two occasions ~ 7 months (IQR 6.7–8.8) apart, 720 pg/ml (95% CI 625–816) vs 670 pg/ml (95% CI 598–796), *P *= 0.5. GDF15 levels were 491 pg/ml in controls (95% CI 429–553), 546 pg/ml (95% CI 478–614) in MS patients, 560 pg/ml (95% CI 502–617) in mild/moderate ME/CFS patients and 602 pg/ml (95% CI 531–674) in severely affected ME/CFS patients. Accounting for potential confounders, severely affected ME/CFS patients had GDF15 concentrations that were significantly increased compared to healthy controls (*P* = 0.01). GDF15 levels were positively correlated (*P *= 0.026) with fatigue scores in ME/CFS.

**Conclusions:**

Severe ME/CFS is associated with increased levels of GDF15, a circulating biomarker of cellular stress that appears which stable over several months.

## Background

Myalgic encephalomyelitis/chronic fatigue syndrome (ME/CFS) is a debilitating condition with an estimated population prevalence of between 0.2 and 2.6% and the most common prevalence estimates in between 0.2 and 0.7% [1–3]. ME/CFS is defined by the presence of unexplained persistent or recurrent fatigue for a period greater than 6 months that results in a reduction in an individual’s ability to maintain previously tolerated levels of occupational, social, educational and personal activity. The activity-limiting symptomatology is heterogeneous and not limited to fatigue [4–6]. The aetiology of ME/CFS remains elusive and although a range of mechanisms have been proposed the main focus of investigation has centred on immune system dysregulation although no consensus exists [7–9]. There is growing interest into the potential contribution of abnormalities in muscle bioenergetics to the phenotype of muscle fatigue [10, 11]. Oxidative stress, mitochondrial dysfunction, acidosis and impaired AMP kinase activity have all been proposed as possible mechanisms contributing to muscle fatigue in ME/CFS [10, 12]. Abnormal fatigue and post-exertional malaise are debilitating symptoms of ME/CFS, which could potentially be explained by abnormalities in energy metabolism. The diagnosis of ME/CFS is based on clinical parameters and as yet there is no biochemical marker for the condition, where attempts to identify disease specific biomarkers have been hampered by the incomplete understanding of the disease pathophysiology. Growth differentiation factor 15 (GDF15) is a circulating peptide which has recently been shown to have a weight lowering effect when administered to rodents and non-human primates [13–15]. A number of observations have led us to speculate as to the role of GDF15 in ME/CFS, considering it as a potential disease biomarker and perhaps a contributor to the symptomatology observed. Firstly, there is emerging evidence that GDF15 has evolved as a signal of cellular stress [16] with growing interest in its role as a biomarker for disorders of mitochondrial function [17]. Secondly, it has also been shown that GDF15 increases following physical activity, which is interesting when considered in the context of post-exertional symptoms described in ME/CFS [18]. Thirdly, in rodent models, increases in circulating GDF15 are reported to be associated with reduced physical activity [19, 20]. To date GDF15 has not been formally studied in the context of ME/CFS. To address this, we measured circulating levels of GDF15 in a clinically and biochemically phenotyped ME/CFS cohort, and compared them with distinct cohorts of healthy individuals and patients with multiple sclerosis (MS), all consenting participants from the UK ME/CFS Biobank (UKMEB) [21].

## Methods

### Participant recruitment

Potential participants of the UK ME/CFS Biobank (UKMEB) were invited from the collaborating National Health System (NHS) primary and secondary care and those who have severe symptoms (being bed- or home-bound) were mainly invited by support groups. The participants received an invitation pack (containing invitation letter from their doctor, information sheet, consent forms, and a questionnaire for initial assessment of symptoms). Once the signed consent form and the symptom assessment questionnaire were received by the research team, a clinical member of the team contacted potential participants for booking appointments, or to explain a possible exclusion at that stage. Participation in the study required participants to attend one of the collaborating clinics, or when not possible, due to severity of disease, they were visited at home by the study research nurse. Participants consented to be reassessed in 6 to 12 months, when all the procedures for clinical assessment, including questionnaires, standardised assessment instruments, blood collections were repeated, as a longitudinal follow-up [22]. The inclusion criteria are: (i) being of 18 years to 60 years old and (ii) having a clinical diagnosis of ME/CFS, according to CDC-1994 [4] or Canadian consensus [5] criteria, confirmed by a clinical researcher after clinical assessment and laboratory tests, the later for differential diagnoses; or (iii) having a confirmed diagnosis of MS given by a consultant neurologist [23] or (iv) being healthy. We excluded from the recruitment those who had: (i) used drugs known to alter immune function (e.g. azathioprine, cyclosporine, methotrexate, steroids), and/or anti-viral medications and vaccinations in the preceding 3 months; (ii) a history of acute and chronic infectious diseases such as hepatitis B and C, tuberculosis, HIV (but not herpes virus or other herpes virus infection); and/or (iii) other severe illness and severe mood disorders. Pregnant women and those within 12 months post-partum and/or currently lactating were also excluded; as well as the healthy individuals who had any history of fatiguing illnesses and/or other conditions that would exclude a diagnosis of ME/CFS (in those with fatigue).

### Clinical assessments

#### Questionnaires

During the appointments for clinical assessment and blood sample collection, an additional extended questionnaire was handed to potential participants containing questions related to: (i) personal and family history, (ii) socio-demographics, (iii) potential risk factors—e.g. smoking and activity levels, among others, and (iv) symptoms in the previous week. The latter included standardised assessment instruments such as Medical Outcomes Survey Short Form—SF-36v2™ [24], for the assessment of functional capacity; the pain analog scale [25], for the assessment of pain severity; and the fatigue severity scale [26], in addition to others not relevant to this paper. Participants were asked to fill in the questions about symptoms and assessment tools within 48 h of blood collection.

#### Anthropometry

The following physical measures were taken at each visit: (i) blood pressure measurements, (Omron HEM-7015IT)—taken at rest in supine and in standing positions, both repeated once; (ii) hand grip strength test (Jamar J00105 hydraulic hand dynamometer)—three repeated 3 s measurements for both hands; (iii) waist and hip circumferences measurements (Wessex non-stretchable sprung tape measure); (iv) standing height (Seca 202 height measure); (v) weight and bioimpedance (Tanita BC-418 MA body composition analyser, including body mass index (BMI)); (vi) spirometry (Vitalograph Pneumotrac 6800 spirometer); (vii) pulse oxymetry (FPX050B finger pulse oximeter);

#### Blood samples collection and analysis

We collected approximately 90–100 ml of blood samples from consenting participants, either at the NHS collaborating services or through home visits (for the severe cases). About 15 ml of blood was used for laboratory tests to exclude differential diagnosis, and approximately 75 ml was processed and stored at the University College London-Royal Free Hospital Biobank (UCL-RFH, https://www.ucl.ac.uk/human-tissue/hta-biobanks/UCL-HTA-licensed-Biobanks) for the planned studies, and for future ethically approved studies. Standard operating procedures (SOPs) were followed, and all the stored aliquots were anonymised. All laboratory blood tests were performed at NHS University Hospitals. GDF15 measurements on participant serum was undertaken at the Cambridge Biochemical Assay Laboratory, University of Cambridge using antibodies & standards from R&D Systems (R&D Systems Europe, Abingdon UK). GDF15 was measured using a microtitre plate-based two-site electrochemiluminescence immunoassay using the MesoScale Discovery assay platform (MSD, Rockville, Maryland, USA). Based on in house data, the assay detection range was 3.0–32,000 pg/ml and between batch imprecision ranging from 6.1 to 9.8%. Serum dilutions demonstrate linearity.

### Statistical analysis

We examined the distribution of GDF15 levels among the study groups, at the two time-points, (means and 95% confidence intervals (95% CI) or media and interquartile range (IQR)). For comparisons between the distinct groups we used t-tests, and for within group comparisons at different time points, we used paired t-test. We used multiple linear regression analyses for inter-group comparisons of GDF15 serum concentration, controlling for potential confounding variables; for the correlations between GDF15 and indicators of severity, including fatigue and pain scores and physical and mental component summaries of the SF-36 instrument, we used simple linear regression. All analyses were done in Stata Version 15.

## Results

### Circulating levels of GDF15 remain stable over time

**Fig. 1:**
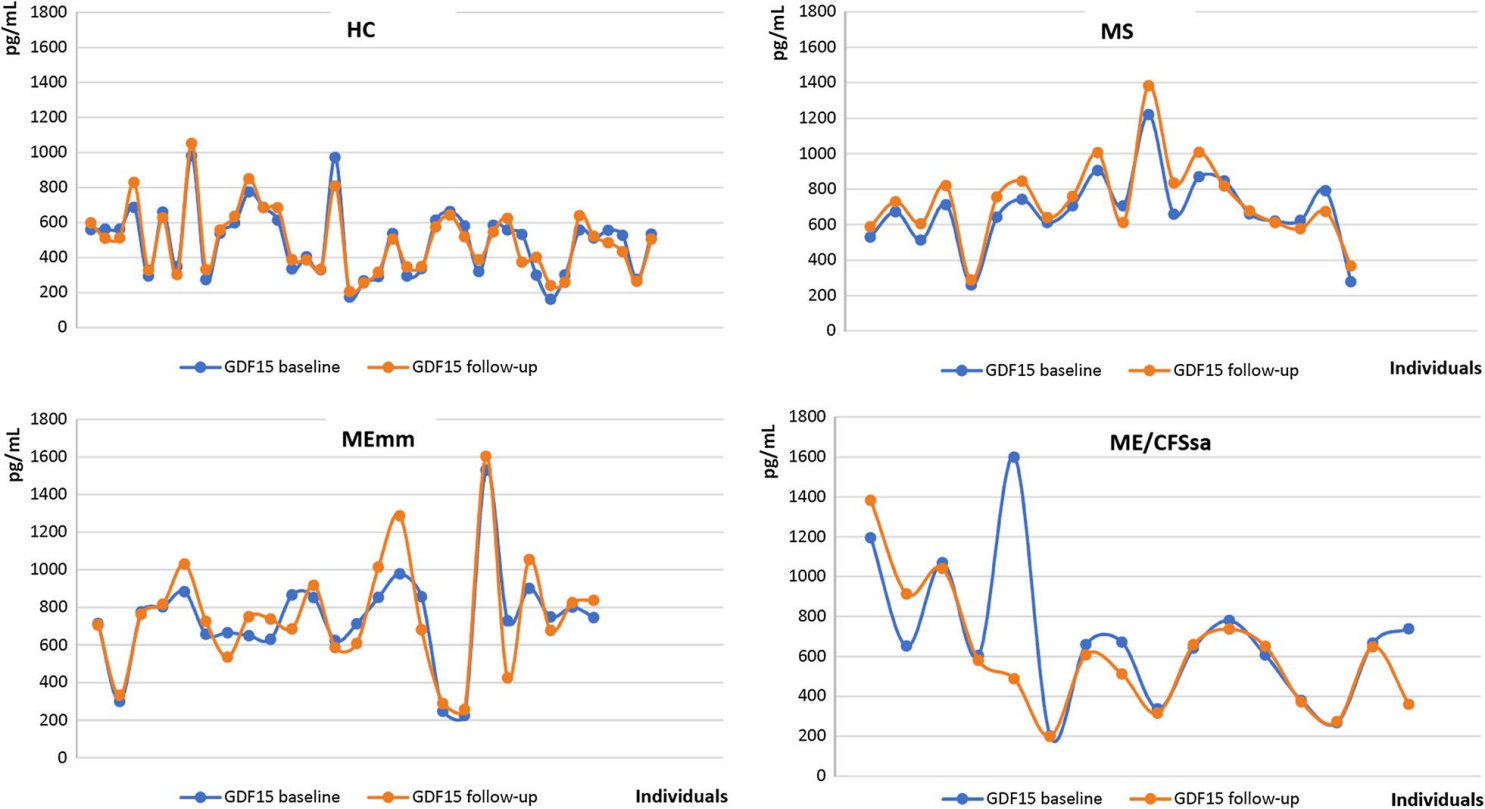
GDF15 levels remain stable at longitudinal follow-up. The plots illustrate the circulating GDF15 levels by each participant at baseline (blue) and follow-up (orange) assessments after a median of 7.4 months (IQR 6.7–8.8). *HC* “healthy controls”, *ME-mm* myalgic encephalomyelitis/chronic fatigue syndrome with mild/moderate symptoms, *ME-sa* myalgic encephalomyelitis/chronic fatigue syndrome severely affected by symptoms, *MS* multiple sclerosis

In considering GDF15 as a potential disease biomarker we wished to examine its stability over time (Fig. 1). We analysed circulating GDF15 at baseline assessment and follow-up in a sub-sample of 100 randomly selected individuals, including 40 healthy volunteers, 40 participants with ME/CFS and 20 MS cases (Table 1). The median time between longitudinal assessments was 7.4 months (IQR: 6.7 to 8.8). In the healthy participants, GDF15 levels remained remarkably stable over time with a mean value at baseline of 491 pg/ml (95% CI 429–553) and follow-up of 495 pg/ml (95% CI 434–556), *p *= 0.7. A similar pattern was observed in ME/CFS cases, where GDF15 values were 720 pg/ml (95% CI 625–816) and 670 pg/ml (95% CI 598–796) at baseline and follow-up respectively (*p *= 0.5). Among participants with MS which represents a progressive neurological illness we observed an increase in the values from baseline to follow-up of with mean circulating GDF15 of 677 pg/ml (95% CI 580–776) and 729 pg/ml (95% CI 619–839) (*p *= 0.01).

**Table 1:** Characteristics of participants with follow-up analysis of GDF-15

All participants (cases and controls)			
Variable	Mean (95% CI) or number (percentage)		
	Healthy control (n = 40)	Multiple sclerosis (n = 20)	ME/CFS (n = 40)
Age (years)**	42.9 (39.1–46.6)	52.7 (49.8–55.6)	44.1 (40.1–48)
BMI (Kg/m^2^)	25.9 (24.4–27.4)	24.7 (23–26.4)	25.6 (23.8–27.4)
eGFR (ml/min/1.73 m^2^)	82.7(79.3–86.1)	75.2 (71–79.5)	79.5 (75.5–83.5)
Female sex	23 (57.5%)	15 (75%)	27 (67.5%)
Current smoking	1 (2.5%)	4 (20%)	4 (10%)

ME/CFS cases		
Variable	Mean (95% CI) or number (percentage)	
	Non-severe cases (n = 24)	Severe cases (n = 16)
Age (years)	47 (42–51.9)	39.8 (33–46.5)
BMI (Kg/m^2^)*	27.1 (24.9–29.3)	22.9 (20.1–25.7)
eGFR (ml/min/1.73 m^2^)**	74.7 (69.4–80)	86.5 (81.8–91.2)
Female sex	16 (66.7%)	11 (68.8%)
Current smoking	3 (12.5%)	1 (6.2%)

*P < 0.05, **P < 0.05

### GDF15 is increased in patients with severe ME/CFS

**Fig. 2:**
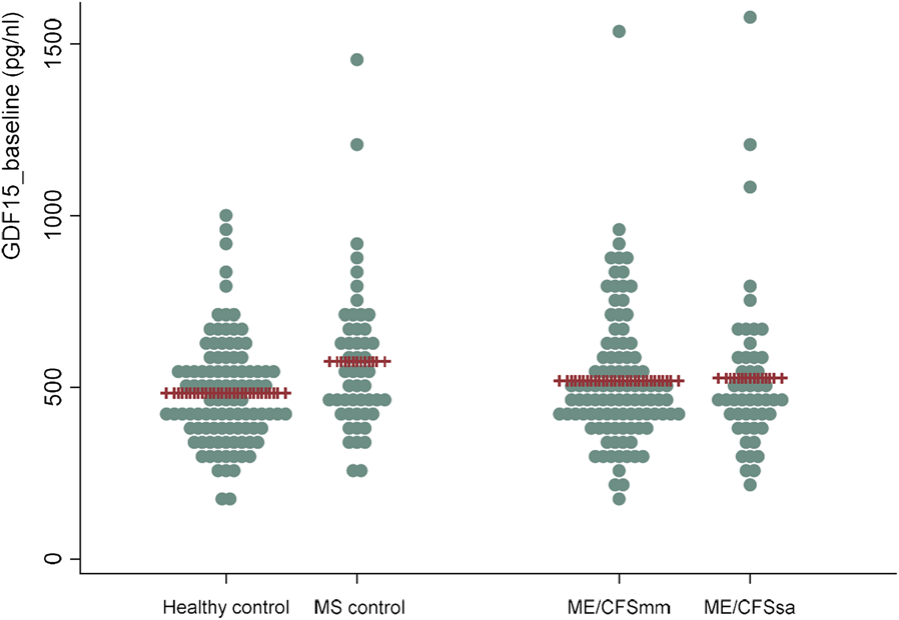
GDF15 levels across case and control populations. The plot illustrates the range of circulating GDF15 levels measured at baseline visits across the four study cohorts. No statistically significant difference in GDF15 was observed between the groups. Mean values are marked in each group. *MS* multiple sclerosis, *ME/CFSmm* myalgic encephalomyelitis/chronic fatigue syndrome with mild/moderate symptoms, *ME/CFSsa* myalgic encephalomyelitis/chronic fatigue syndrome severely affected by symptoms

Table 2 shows baseline characteristics of the study population, this included cases of ME/CFS (n = 150) categorised as either mild/moderate disease (n = 100) and severely affected (i.e. housebound or bedbound, n = 50), MS (n = 50) and “healthy controls” (n = 100). The MS cohort were included as a comparative group where patients also had a chronic disease associated with fatigue. People with MS were on average older (49 years old), compared to both ME/CFS and “healthy controls” (mean age ~ 42 years old). A larger proportion of those with MS smoked (26%), compared to 7.5% of ME/CFS and 4% of healthy controls. When comparing severe with non-severe cases of ME/CFS, we found a lower BMI and higher eGFR in those with more severe disease. Other parameters analysed were similar among groups. The mean baseline GDF15 values were 491 pg/ml (95% CI 429–553) in healthy controls, 546 pg/ml (95% CI 478–614) in those with MS, 560 pg/ml (95% CI 502–617) in those with mild/moderate ME/CFS and 602 pg/ml (95% CI 531–674) in severely affected ME/CFS patients. There was no significant difference between the groups on univariate analysis (Fig. 2). It is well established that GDF15 is increased in a number of physiological and pathological states [17]. Thus, we carried out a multivariate analysis to assess if there were potential differences in GDF15 levels across the groups, after controlling for independent variables, such as: sex, age, body mass index, activity levels, smoking status, estimated Glomerular Filtration Rate (eGFR) and C-reactive protein (CRP)—a frequently used circulating marker of inflammation. We found a significant direct association between the independent variables: age (years), BMI, and CRP, and GDF15 levels; and an inverse association between the GDF15 levels and eGFR (Table 3). The multivariate analysis results show that cases of severe ME/CFS (but not of mild/moderate cases) have values of GDF15 that are significantly higher than the healthy group (*P *= 0.01). There were no significant differences in GDF15 between ME/CFS cases with mild/moderate disease, MS cases, and healthy volunteers (Table 3).

**Table 2:** Participant characteristics

All groups			
Variable	Mean (95% CI) or number (percentage)		
	Healthy control (n = 150)	Multiple sclerosis (n = 50)	ME/CFS (n = 150)
Age (years)**	42 (39.7–44.3)	49.4 (43.3–51.4)	41.6 (39.8–43.5)
BMI (Kg/m^2^)	25.9 (24.9–26.8)	25.5 (24.3–26.8)	25.4 (24.6–26.3)
eGFR (ml/min/1.73 m^2^)	82.6 (80.7–84.6)	80.6 (77.9–83.3)	82.5 (80.9–84.2)
C-reactive protein(mg/l)	3.5(2.1–5.0)	2.4(1.8–2.9)	2.8(2.2–3.4)
Female sex	65 (65%)	38 (76%)	112 (74.6%)
Current smoking**	4 (4%)	13 (26%)	11 (7.5%)

ME/CFS cases		
Variable	Mean (95% CI) or number (percentage)	
	Non-severe cases (n = 100)	Severe cases (n = 50)
Age (years)	41.2 (38.9–43.4)	42.6 (39.4–45.7)
BMI (Kg/m^2^)*	26.2 (25.2–27.3)	23.7 (22.4–25.0)
eGFR (ml/min/1.73 m^2^)*	80.5 (78.3–86.2)	86.7 (84.7–88.6)
C-reactive protein(mg/l)	3.1(2.4–3.8)	2.2(1.4–3.1)
Female sex	74 (74%)	38 (76%)
Current smoking	10 (10.3%)	1 (2%)

*P < 0.01; **P < 0.001

**Table 3:** Multivariate linear regression on the effects of being a case on GDF15 concentrations

Variable	Coefficient^a^ (pg/ml)	95% confidence interval	p-value^b^
Case status			0.08
Healthy control	–		
MS	34.3	− 33.7 to 102.4	0.3
ME/CFS mild/moderate	48.3	− 9.0 to 105.6	0.1
ME/CFS severe	91.2	19.6 to 162.7	*0.01*
Sex (female)	− 28.5	− 73.7 to 16.8	0.2
Age^c^ (years)	3.9	1.8 to 6.1	*< 0.0001*
Body mass index (Kg/m^2^)^c^	3.3	− 1.4 to 8.0	0.2
Activity level			0.05
Little activity	–		
Average activity	66.7	7.2 to 126.3	*0.03*
Active	45.2	− 8.7 to 99.1	0.1
Smoking status			*0.04*
Never smoked	–		
Previous smoker	7.7	− 38.0 to 53.3	0.7
Current smoker	92.6	19.2 to 165.9	*0.01*
CRP (mg/L)	6.9	2.5 to 11.2	*0.002*
eGFR(ml/min/1.73 m^2^)	− 4.0	− 6.3 to − 1.6	*0.001*

*p*-value < 0.05 was considered statistically significant and represented in italics

^a^Difference between GDF15 concentration in exposure group and baseline variable. Baseline concentration in GDF15 in “healthy controls” with baseline levels of exposure = 511.5 pg/ml

^b^Log-likelihood test

^c^By unit of change, e.g. year of age

### Circulating GDF15 is associated with self-reported fatigue

**Fig. 3:**
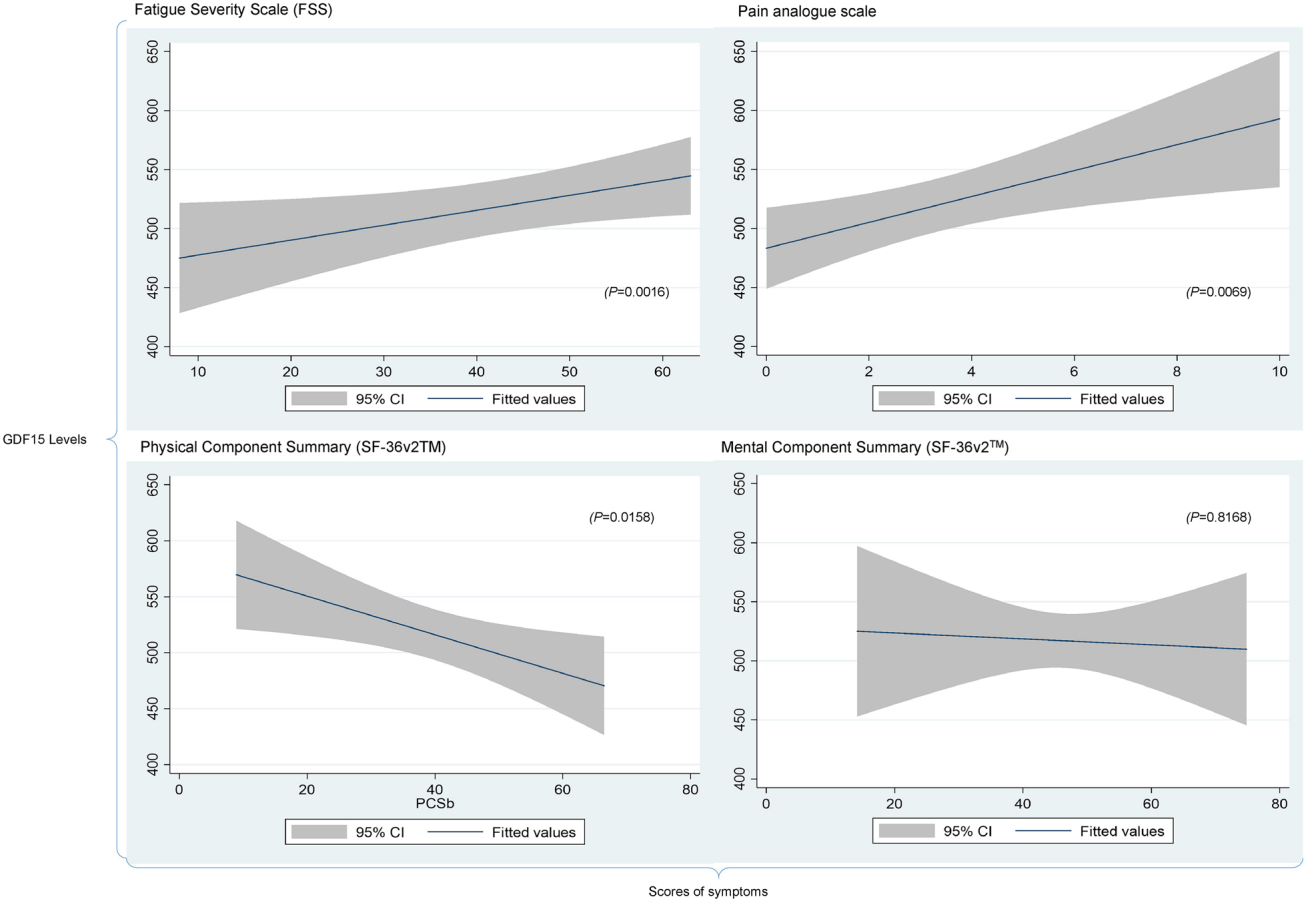
Linear regression charts between levels of GDF15 and severity of symptoms reported by study participants at baseline time-point. The charts illustrate the regression lines between levels of circulating GDF15 (y-axis) and reported symptoms (x-axis) in all participants (healthy control, Multiple Sclerosis and ME/CFS (mild and severe disease). Symptoms measured by distinct validated instruments, at the time of blood collection (baseline time-point) and 95% Confidence Intervals. The statistical significance of the tested correlations between levels of GDF15 and measured symptoms are also presented in the charts (*P* values)

**Fig. 4:**
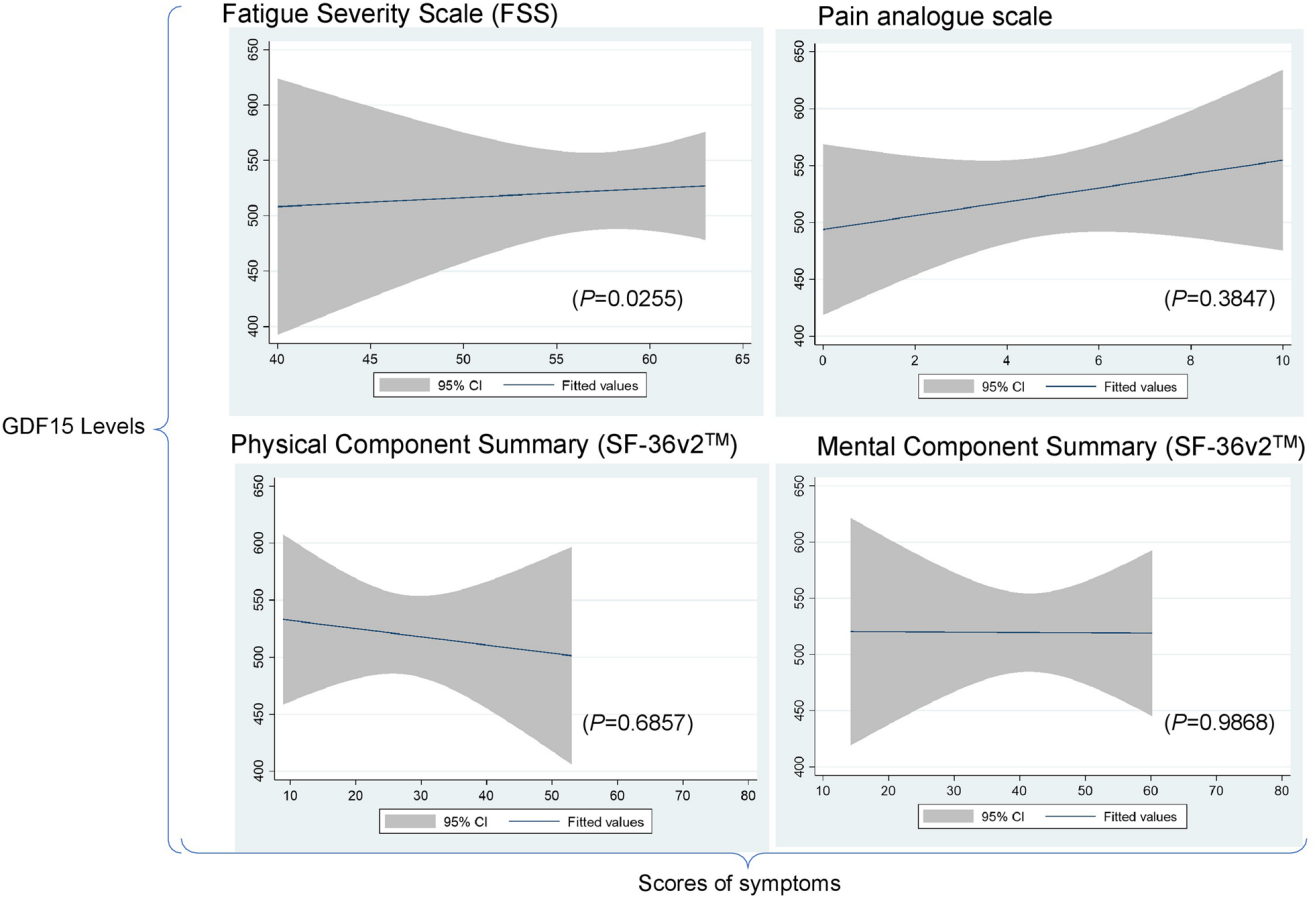
Linear regression charts between levels of GDF15 and severity of symptoms reported by study participants with ME/CFS at baseline time-point. The charts illustrate the regression lines between levels of circulating GDF15 (y-axis) and reported symptoms (x-axis) among the ME/CFS patient cohort only, including participants in the mild and severe disease categories. Symptoms were measured by distinct validated instruments, at the time of blood collection (baseline time-point) and 95% Confidence Intervals. The statistical significance of the tested correlations between levels of GDF15 and measured symptoms are also presented in the charts (*P* values)

To further delineate the relationship between GDF15 and symptom severity we examined the correlation between circulating GDF15 levels and a variety of self-reported severity outcomes in our cases and control cohort (Table 2). These included the fatigue severity scale (FSS), pain analog scale and the normalised physical and mental summary scores from SF-36v2™ instrument. The FSS scores vary from 9 (no fatigue) to 63 (very severe fatigue). The pain analog scale allows scores varying from 0 to 100, with “0” representing absence of the symptoms and “100” corresponding to extreme pain. For the SF-36v2™ summary scores, the lower the normalised scores, the worse is quality of life in relation to physical and mental domains. Table 4 shows the distribution of severity scores of fatigue, pain, and SF-36v2™ physical and mental component summaries for all groups. Figure 3 illustrates the direct correlation between both fatigue and pain intensity and GDF15 concentrations in all of the cases of ME/CFS, MS, and control subjects. An inverse relationship between physical wellbeing and GDF15 was present, where a lower score on the SF-36v2 ™ indicates reduced physical health. In this cohort we did not see a significant association between GDF15 and the SF-36v2 ™ mental health summary score. The regression lines and 95% Confidence Intervals show in the charts indicates the impact of changes in the levels of GDF15 in the symptoms’ severity scores or vice versa. In the subset of ME/CFS participants studies we observed a direct correlation between fatigue and GDF15 levels (Fig. 4).

**Table 4:** Mean, median and spread of outcome measurements by participant groups

Instrument	Healthy controls				Multiple sclerosis				ME/CFS mild/moderate				ME/CFS severe symptoms			
	Mean	SD	Median	IQR	Mean	SD	Median	IQR	Mean	SD	Median	IQR	Mean	SD	Median	IQR
FSS	20.1	9.22	18	14–25	46.7	14.3	51	37–58	57.3	6.7	59	54–63	59.2	3.5	60	57–62
PAS	0.96	1.46	0.5	0–1.2	3.4	2.7	3	0.7–6.2	4.9	2.4	5.3	3.1–6.9	5.4	2.6	6	3.2–7.5
PCS	57.1	4.9	58.1	55.9–60.3	38.3	12.3	36.9	28.5–48.3	39.1	9.9	40.4	32.6–46.3	19.1	4.8	19.8	15.6–22.2
MCS	52.1	8.1	54.9	49.6–57.6	46.0	10.8	47.5	38.5–54.6	59.2	3.6	60	57–62	44.0	9.9	45.6	38.4–51.1

*FSS* Fatigue Severity Scale, *PAS* Pain Analog Scale, *PCS* Physical Component Score (SF-36v2™), *MCS* Mental Component Score (SF-36v2™), *SD* standard deviation, *IQR* interquartile range, *ME/CFS* myalgic encephalomyelitis/chronic fatigue syndrome

## Discussion

GDF15 was initially isolated as a transcript produced by activated macrophages, which encoded a protein with some homology to the transforming growth factor beta (TGFβ) superfamily [27]. Increased circulating GDF15 levels have been observed following physical activity and during pregnancy with increases as much as 40-fold in the latter [18, 28]. Elevations in GDF15 are not limited to these physiological states and in the 20 years since its discovery, increases in the peptide have been reported in a variety of pathological conditions including aging, cardiac failure, chronic kidney disease, mitochondrial disease and malignancy [16, 17]. However, it was the identification of GFRAL, a transmembrane receptor localised to the hindbrain, as the putative target for GDF15 and mediator of its weight lowering effects in rodents that framed GDF15 as a potential anti-obesity therapy [13–15].

As the study of the weight lowering effects of GDF15 continues it has been intriguing to see that GDF15 is not regulated by the nutritional stimuli known to influence other hormones implicit in energy homeostasis, this suggests that GDF15 has not evolved for this biological purpose [16, 29]. The spectrum of conditions reported to be associated with elevations in GDF15 share a common thread of cellular stress, raising the possibility that GDF15 may have evolved as part of a stress response signal that may incidentally influence energy balance. Insights into the role of GDF15 as a signal of cellular stress has been aided by the study of disorders of mitochondrial function. Recent work from Chung et al. illustrate that in mice with a muscle specific knockout for *crif1*, a protein integral to the mitoribosomal subunit 39S, the ensuing mitochondrial unfolded protein response mediates increased GDF15 expression in a process that is dependent on the activation of the CHOP, a stress induced pro-apoptotic transcription factor [30]. Similarly, in vitro, GDF15 expression is responsive to activation of the integrated stress response (IRS), an adaptive response to stressful stimulus in eukaryotic cells [29, 31]. Markedly increased circulating GDF15 values have now been reported in a variety of inherited mitochondrial diseases [32–35]. The apparent symptom overlap between patients affected by ME/CFS and mitochondrial disorders has generated discussion into the possibility of a shared pathophysiology between the respective conditions [36]. Considering GDF15’s potential as a novel marker of mitochondrial dysfunction, we hypothesised that it may represent a biomarker of mitochondrial stress in ME/CFS [37].

In our analysis, we included both healthy control subjects in addition to a cohort of patients diagnosed with multiple sclerosis. The observation that GDF15 measurements do not significantly change over longitudinal follow-up of patients affected with ME/CFS reassures us of its value as a potential biomarker. Interestingly in the subset of participants with MS we saw that GDF15 increased significantly from baseline assessment. GDF15 levels are significantly increased in a cohort of ME/CFS patients categorised as having severe disease when compared to a healthy control group. The observation that GDF15 levels associate with severe rather than mild/moderate disease is indicative of a relationship between ME/CFS disease severity and GDF15. These findings mirror reports from the study of primary mitochondrial disorders with Yatsuga et al. [34] demonstrating a positive correlation between GDF15 and both the Japanese Mitochondrial Disease Rating Scale and the Newcastle Mitochondrial Scale for Adults, which represent semi-quantitative clinical rating scales. In the case of our ME/CFS study cohort the fatigue severity scale correlated with GDF15 levels, these findings lend further support for a role for GDF15 as a marker of symptom severity in ME/CFS.

However, it is important to note that in contrast to the levels observed in primary disorders of mitochondrial function, the mean GDF15 levels measured in our ME/CFS cohort are many fold lower [33, 35]. This is not surprising given the widespread mitochondrial dysfunction observed in disorders such as Leigh’s syndrome, but it suggests that significant impairment of mitochondrial function may not be as prominent a feature of ME/CFS. Certainly, enrichment for pathogenic mutations in mitochondrial DNA has not been seen among patients affected by ME/CFS [38]. However, there are a number of conflicting reports on the relationship between mitochondrial dysfunction and disease severity in ME/CFS [12]. We expect that the emergence of GDF15 as a biomarker of mitochondrial dysfunction will support ongoing efforts to study this relationship.

In ME/CFS it is not clear what tissue or tissues may be contributing to the elevated levels GDF15 observed in severe disease. We know that in humans, GDF15 is expressed in a wide range of tissues with highest expression in the liver, kidney, prostate, colon, adipose tissue and placenta [39]. Interestingly, GDF15 or MIC-1 was initially cloned from a macrophage cell line. However, Sweetman et al. [40] did not report a significant change in GDF15 transcription in PBMCs from ME/CFS patients. We have seen in primary mitochondrial disorders associated with overt myopathy that GDF15 expression is massively upregulated in skeletal muscle and this is associated with an increase in circulating levels [32]. However, there are no reports of GDF15 expression in muscle or other tissues of humans with ME/CFS. Further study is required to determine the source of increased circulating GDF15 observed in a subset of ME/CFS patients.

Thus far we have considered GDF15 as a potential biomarker in ME/CFS rather than a contributing factor to the symptomatology observed in this cohort. Fatigue is frequently reported in the conditions associated with increased GDF15, although the relationship between fatigue in these disease states and GDF15 has not been formally studied in humans. Reduced locomotor activity has been reported in transgenic mice that over-express GDF15 and wild-type animals exposed to pharmacological doses of the peptide [19, 20]. The reason for the decreased activity has not been elucidated, however, it is speculated that this may be reflective of reduced food seeking behaviour. In humans, understanding whether GDF15 contributes to the symptoms of fatigue or is simply a circulating marker of the underlying disease process will necessitate further study. However, in time, both the administration of GDF15 to humans and/or the pharmacological manipulation of the GDF15-GFRAL axis in specific diseases that increase GDF15 could provide valuable insight on the contribution of GDF15 to symptoms of fatigue. Certainly, in ME/CFS the biological relevance of the circulating levels at which GDF15 we have observed is unclear.

## Conclusions

We have demonstrated that under resting conditions circulating GDF15 is increased among patients with severe ME/CFS and levels of GDF15 remain stable over a period of months. Further study is required to establish the tissue/tissues contributing to the elevations in GDF15, and the mechanism by which it is increased in ME/CFS. It will also be important to investigate whether an elevation in GDF15 levels could contribute to any of the symptomatology of ME/CFS, even if only in a subset of patients.

## Data Availability

The datasets generated during and/or analysed during the current study are available from the corresponding author on reasonable request.

## Abbreviations

BMI: body mass index
CDC: centres for disease control and prevention
CI: confidence interval
CRP: C-reactive protein
eGFR: estimated glomerular filtration rate
GDF15: growth differentiation factor 15
HIV: human immunodeficiency virus
IQR: interquartile range
ME/CFS: myalgic encephalomyelitis/chronic fatigue syndrome
MIC-1: macrophage inhibitory factor 1
MS: multiple sclerosis
PBMC: peripheral blood mononuclear cell
TGFβ: transforming growth factor beta
